# Overexpression of MAPK15 in gastric cancer is associated with copy number gain and contributes to the stability of c-Jun

**DOI:** 10.18632/oncotarget.4171

**Published:** 2015-05-19

**Authors:** Dong-Hao Jin, Jeeyun Lee, Kyoung Mee Kim, Sung Kim, Duk-Hwan Kim, Joobae Park

**Affiliations:** ^1^ Department of Molecular Cell Biology, Samsung Biomedical Research Institute, Sungkyunkwan University School of Medicine, Suwon, Korea; ^2^ Department of Internal Medicine, Samsung Medical Center, Sungkyunkwan University School of Medicine, Seoul, Korea; ^3^ Department of Pathology and Translational Genomics, Samsung Medical Center, Sungkyunkwan University School of Medicine, Seoul, Korea; ^4^ Department of Surgery, Samsung Medical Center, Sungkyunkwan University School of Medicine, Seoul, Korea

**Keywords:** gastric cancer, copy number alteration, MAPK15, c-Jun, stability

## Abstract

This study was aimed at understanding the functional and clinicopathological significance of *MAPK15* alteration in gastric cancer. Genome-wide copy number alterations (CNAs) were first investigated in 40 gastric cancers using Agilent aCGH-244K or aCGH-400K, and copy number gains of *MAPK15* found in aCGH were validated in another set of 48 gastric cancer tissues. The expression of MAPK15 was analyzed using immunohistochemistry in concurrent lesions of normal, adenoma, and carcinoma from additional 45 gastric cancer patients. The effects of MAPK15 on cell cycle, c-Jun phosphorylation, and mRNA stability were analyzed in gastric cancer cells. Copy number gains of *MAPK15* were found in 15 (17%) of 88 tumor tissues. The mRNA levels of *MAPK15* were relatively high in the gastric cancer tissues and gastric cancer cells with higher copy number gains than those without. Knockdown of *MAPK15* using siRNA in gastric cancer cells significantly suppressed cell proliferation and resulted in cell cycle arrest at G_1_-S phase. Reduced c-Jun phosphorylation and c-Jun half-life were observed in *MAPK15*-knockdowned cells. In addition, transient transfection of *MAPK15* into AGS gastric cancer cells with low copy number resulted in an increase of c-Jun phosphorylation and stability. The overexpression of MAPK15 occurred at a high frequency in carcinomas (37%) compared to concurrent normal tissues (2%) and adenomas (21%). In conclusion, the present study suggests that MAPK15 overexpression may contribute to the malignant transformation of gastric mucosa by prolonging the stability of c-Jun. And, patients with copy number gain of MAPK15 in normal or premalignant tissues of stomach may have a chance to progress to invasive cancer.

## INTRODUCTION

Gastric cancer is the third leading cause of cancer deaths worldwide despite a rapid decline in its incidence over the recent few decades. The past few years have witnessed major advances in the early detection and multi-modality therapeutic approach in patients with gastric cancer. Nonetheless, approximately half of the patients who undergo curative surgical resection still develop loco-regional or distant metastases and die from the disease. Five-year survival rates of gastric cancer patients reach below 30% in most countries [1]. Accordingly, it is important to identify new biomarkers for the early detection and targeted therapy of patients with gastric cancer.

DNA copy number alteration (CNA) defined as DNA segments that are 1 kb or larger in size, is an important type of genetic alteration observed in cancer cells [2]. Variance of gene expression in CNAs may be greater than elsewhere in the genome. The variation in expression of genes within CNAs is partly due to altered gene dosage [3]. Recently, we investigated CNAs in 40 gastric cancers using array comparative genomic hybridization (aCGH) and identified frequent copy number gains at 8q24.3. Several other studies have also revealed high-level copy number gains at 8q24 in gastric cancers [4-7]. A number of genes (*PLEC1, GPAA1, SHARPIN, BOP1, HSF1, SLC39A4, RECQL*4) in this region were reported to play an oncogenic role [8-14], whereas genes such as *SCRIB, PUF60*, and *PARP10* were reported to play a suppressive role in the tumorigenesis of human cancers [15-17].

*MAPK15* at 8q24.3, also known as extracellular signal-regulated kinase (ERK) 8 for human or ERK7 for mouse or rat protein, is activated by serum and a Src-dependent signaling pathway. MAPK15 has been proposed as an atypical MAP kinase based on the absence of specific MEKs upstream, making it different from typical MAPKs such as ERK1/2, JNK, p38s, and ERK5 [18-20]. MAPK15 is expressed at high levels in anaplastic thyroid carcinoma cells, and can be activated by RET/PTC3, an activated form of the RET proto-oncogene [21, 22]. MAPK15 maintains genomic integrity by inhibiting HDM2-mediated PCNA degradation [23] and increases tumorigenesis of human colon cancer by c-Jun activation [24]. Additionally, MAPK15 is also known to modulate telomerase activity at least in part by regulating hTERT mRNA expression [25]. All these data suggest that MAPK15 may play an important role in the development of human cancer and is an attractive target for cancer therapy. However, the role of MAPK15 on the development of gastric cancer remains to be elucidated.

To further understand the clinicopathological significance of MAPK15 and the mechanism underlying its oncogenic role in gastric cancer, we analyzed the effect of *MAPK15* knockdown or overexpression on cell cycle, c-Jun phosphorylation, and c-Jun stability in gastric cancer cells. Furthermore, we investigated MAPK15 protein levels in concurrent lesions of normal, adenoma and carcinoma tissues from gastric cancer patients.

## RESULTS

### Copy number alterations of MAPK15 in gastric cancer

**Figure 1:**
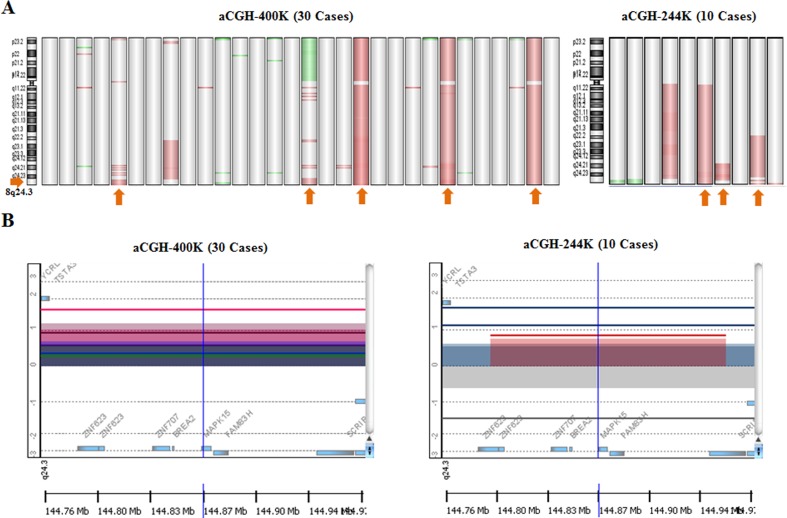
DNA copy number alterations (CNAs) on chromosome 8 **A.** The CNAs in 30 gastric cancers were analysed by agilent aCGH-400K and 10 by aCGH-244K. The diagram shows CNAs on chromosome 8 detected by aCGH-400K (left) and aCGH-244K (right). Vertical lines represent cytoband of chromosome 8. The red and green colors indicate regions of DNA copy number gains and losses, respectively. Arrows indicate samples with copy number gains of MAPK15. **B.** Aberrations around *MAPK15* gene at 8q24.3 are shown. Vertical lines indicate log_2_-based intensity ratios values, and each colored horizontal line represents a copy number alteration. Log2 ratios of signal intensities of samples with normal copy number are plotted with the horizontal central line equal to zero. Horizontal lines above the 0 of log_2_-based intensity ratio in the aCGH-400K and aCGH-244K indicate samples with 8q24.3 amplification. Five horizontal lines above the zero in aCGH-400K and three horizontal lines in aCGH-244K indicate samples with copy number gains.

**Figure 2:**
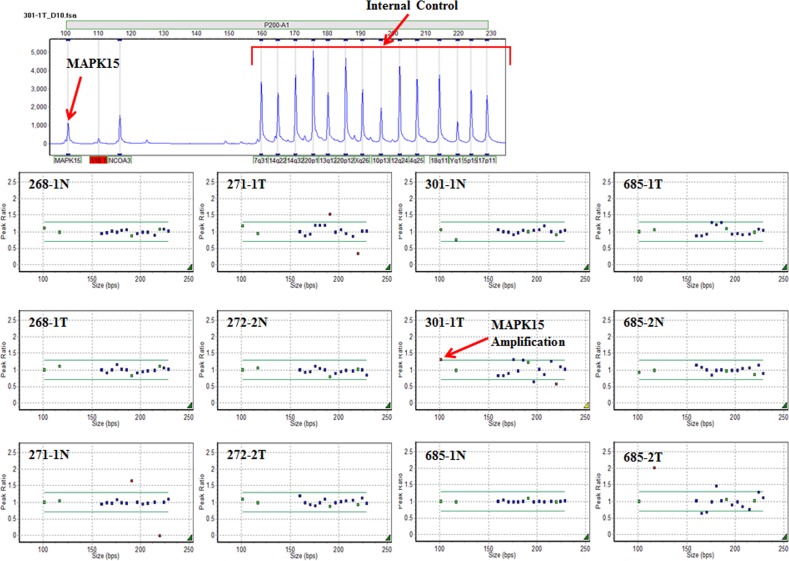
Multiplex ligation-dependent probe amplification (MLPA) of *MAPK15* Upper panel shows a representative image of capillary electrophoresis signals analyzed by MLPA and GeneMaker 2.0.0. Lower panel indicates DNA copy number of *MAPK15* detected by MLPA in 6 paired samples of gastric cancer matched with the normal (268-1, 271-1, 272-2, 301-1, 685-1 and 685-2). Probe ratios below 0.7 and above 1.3 indicate loss and gain, respectively. The “T” and “N” represent tumor and normal tissues, respectively.

Tissues from 133 gastric cancer patients were analyzed in this study: 40 for aCGH, 48 for the validation of aCGH, and 45 for immunohistochemistry. We first investigated genome-wide copy number alterations (CNAs) in 40 gastric cancers using Agilent aCGH-244K or aCGH-400K and identified copy number gains (20%) on 8q24.3 where *MAPK15* is located (Figure 1). We validated the CNAs of *MAPK15* obtained from the aCGH. DNA copy number of *MAPK15* in six samples (268-1, 271-1, 272-2, 301-1, 685-1 and 685-2) with available tumor and matched normal tissues among the 40 samples was analyzed by multiplex ligation-dependent probe amplification (MLPA) (Figure 2). The peak ratio of *MAPK15* in 301-1T was above 1.3 and the others were within the normal copy number range between 0.7 and 1.3. These MLPA-based data supported the CNAs identified by the aCGH.

### Correlation between MAPK15 copy number and expression

**Figure 3:**
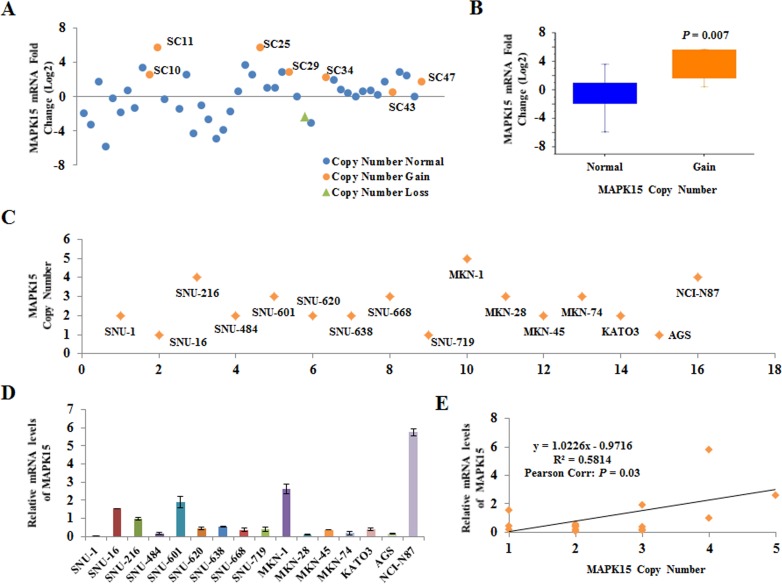
Relationship between copy number alterations and mRNA levels **A.** *MAPK15* mRNA levels in 48 gastric cancers with matched normal tissues were analyzed by qRT-PCR. The mRNA level in each sample was normalized to the internal control of RPLP0, and fold change of *MAPK15* mRNA levels was calculated as a ratio of tumor tissue to matched normal tissue. The baseline value 0 corresponds to a fold change of 1. Blue, orange, and green colors indicate samples without *MAPK15* copy number change, with copy number gain, and copy number loss, respectively. **B.** The fold changes of *MAPK15* mRNA in cancers with or without *MAPK15* copy number alteration was compared using one sample t-test (*P* = 0.007). **C.** *MAPK15* copy number in 16 gastric cancer cell lines was analyzed by qPCR. **D.** *MAPK15* mRNA levels in 16 gastric cancer cell lines were measured by qRT-PCR. The mRNA level in each sample was normalized to the internal control of GAPDH. The average mRNA level in the 16 cell lines was considered as value 1, and relative values were calculated. Error bars indicate standard deviation (*n* = 3). **E.** Correlations between *MAPK15* copy numbers and its mRNA levels in 16 gastric cancer cell lines were analyzed by the Pearson's correlation coefficients (*P* = 0.03).

We next analyzed the CNAs and mRNA levels of *MAPK15* in another set of 48 fresh-frozen tumor and matched normal tissues and in 16 gastric cancer cell lines using qPCR to validate the aCGH results and understand the correlation between copy number of *MAPK15* and its expression. We found there were no copy number gains or losses in 48 normal tissues, but 7 (15%) of 48 tumor tissues represented copy number gains, and 1 (2%) showed a loss (Supplementary Figure 1). The mRNA levels of *MAPK15* in 48 gastric cancer tissues were detected by qPCR (Supplementary Figure 2) and their associations with copy number were analyzed (Figure 3A and 3B). The mRNA levels of *MAPK15* in gastric cancer were significantly different between tissues with CNAs and those without (*P* = 0.007). In this study, 17 samples showed high mRNA levels of *MAPK15* without copy number alteration (Figure 3A), suggesting that MAPK15 overexpression may result from other molecular alterations in addition to CNAs in gastric cancer.

The copy number of *MAPK15* was gained in 7 (44%) cell lines (MKN-1, MKN-28, MKN-74, NCI-N87, SNU-216, SNU-601 and SNU-668) and was lost in 3 (19%) cell lines (AGS, SNU-16 and SNU-719) (Figure 3C). *MAPK15* mRNA levels were significantly high in cell lines (MKN-1, NCI-N87, SNU-216 and SNU-601) with copy number gains (Figure 3D). There was a high correlation between the copy number and mRNA levels of *MAPK15* (R^2^ = 0.58, *P* = 0.03; Figure 3E). We also measured protein levels of MAPK15 in the 16 gastric cancer cell lines. No significant correlation between *MAPK15* mRNA and protein levels was observed (Supplementary Figure 3). Based on these observations, it is likely that *MAPK15* amplification may be common in gastric cancer and may influence the mRNA levels of *MAPK15*. However, MAPK15 expression *in vitro* may be controlled at the level of post-transcription or post-translation.

### Knockdown of MAPK15 inhibits gastric cancer cell proliferation

**Figure 4:**
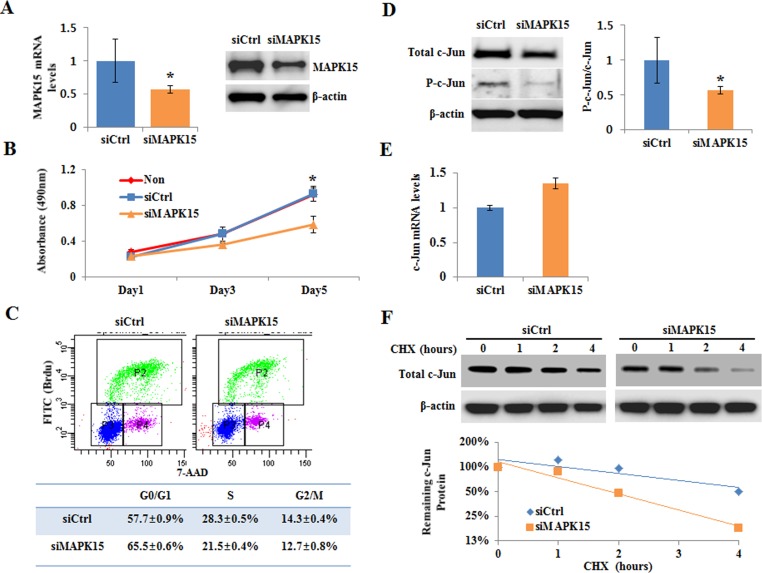
The effect of *MAPK15* knockdown on cell cycle and c-Jun stability The SNU-601 cells were transfected with MAPK15 siRNA (siMAPK15) or nonspecific siRNA (siCtrl). **A.** The mRNA and protein levels of MAPK15 were measured using qRT-PCR and immunoblot analysis, respectively, on the 3^rd^ day after siRNA transfection. Error bars indicate standard deviation (*n* = 3, *P < 0.05). **B.** Cell proliferation was detected by MTS assay. Absorbance at 490 nm was measured on the 1^st^, 3^rd^, and 5^th^ day after siRNA transfection. Error bars indicate standard deviation (*n* = 4, *P < 0.05). **C.** On the 3^rd^ day of post-siRNA transfection, cells were treated with 10 μM BrdU for 2 hours, and collected. The cells were incubated with a FITC-conjugated anti-BrdU antibody. Total DNA was stained with 7-AAD. **D.** The protein levels of c-Jun and P-c-Jun were detected by immunoblot analysis on the 3^rd^ day after siRNA transfection. *MAPK15* knockdown experiment was performed three times, and the ratio of phosphor-c-Jun to total c-Jun was found to be significantly decreased in cells transfected with siMAPK15 than in those transfected with siCtrl (*P* = 0.02, Wilcoxon rank-sum test). **E.** The mRNA levels of c-Jun were detected by qPCR on the 3^rd^ day after siRNA transfection. **F.** On the 3^rd^ day after siRNA transfection, cells were treated with cycloheximide (80μg/ml), a protein synthesis inhibitor, for 0, 1, 2, or 4 hours, and the c-Jun protein level was analyzed by immunoblotting. The experiment was performed twice, and a similar result was obtained.

To understand the biological function of MAPK15 in gastric cancer, we analyzed the effects of *MAPK15* downregulation on cell proliferation and cell cycle in gastric cancer cells. Three different sequences of siMAPK15 (BioNeer, DaeJeon, Korea) for *MAPK15* knockdown were validated in four cell lines according to RNA interference guidelines. *MAPK15* mRNA levels were found to be suppressed by all sequences (Supplementary Figure 4), and the siMAPK15#2 was used in subsequent experiments for *MAPK15* knockdown. SNU-601 cells with the copy number gain and increased expression of MAPK15 were transfected with siRNA of MAPK15 (siMAPK15) or nonspecific siRNA (siCtrl). The mRNA and protein levels of *MAPK15* were found to be substantially suppressed by transfection of siMAPK15 (Figures 4A). Knockdown of *MAPK15* resulted in inhibition of cell proliferation and in cell cycle arrest at the G_1_-S phase. The absorbance increased gradually with time but was significantly lower in siMAPK15 than in siCtrl on the 5^th^ day post-transfection (Figure 4B). Cell cycle was analyzed using BrdU FITC assay (Figure 4C) on the 3^rd^ day after siMAPK15 transfection in SNU-601 cells. The assay showed that G_1_ to S phase transition was significantly inhibited by *MAPK15* knockdown: the fraction of cells in G_0_/G_1_ increased approximately 7∼8% in cells transfected with siMAPK15 compared to control cells (*P* = 0.04, Wilcoxon rank-sum test).

### MAPK15 as a protector of c-Jun in gastric cancer

We further examined the effect of MAPK15 on c-Jun activation in gastric cancer cells to understand the molecular mechanisms underlying G_1_-S arrest by *MAPK15* knockdown. Immunoblot analysis revealed that the levels of total c-Jun and phosphorylated-c-Jun (P-c-Jun) proteins were substantially decreased in cells transfected with siMAPK15 than in those transfected with control siCtrl (Figure 4D). To verify that phosphorylated-c-Jun actually decreased under this condition, we calculated the ratio of phosphorylated-c-Jun to total c-Jun and found that the ratio significantly decreased in cells transfected with siMAPK15 than in those transfected with siCtrl (*P* = 0.02, Wilcoxon rank-sum test), suggesting that the decrease in c-Jun phosphorylation is not the consequence of the decrease in c-Jun protein levels. We next determined whether the decreased c-Jun protein level was due to reduced c-Jun transcription in cells transfected with siMAPK15. The mRNA level of c-Jun, however, was higher in cells transfected with siMAPK15 than in those transfected with siCtrl (Figure 4E). These results suggest that low c-Jun abundance in cells transfected with siMAPK15 might be associated with the stability of c-Jun protein. We further examined the half-life of c-Jun protein in cells transfected with siMAPK15 or siCtrl after treatment with the protein synthesis inhibitor, cycloheximide (80μg/ml) (Figure 4F). The half-life of c-Jun was shorter in cells transfected with siMAPK15 than in those transfected with siCtrl.

**Figure 5:**
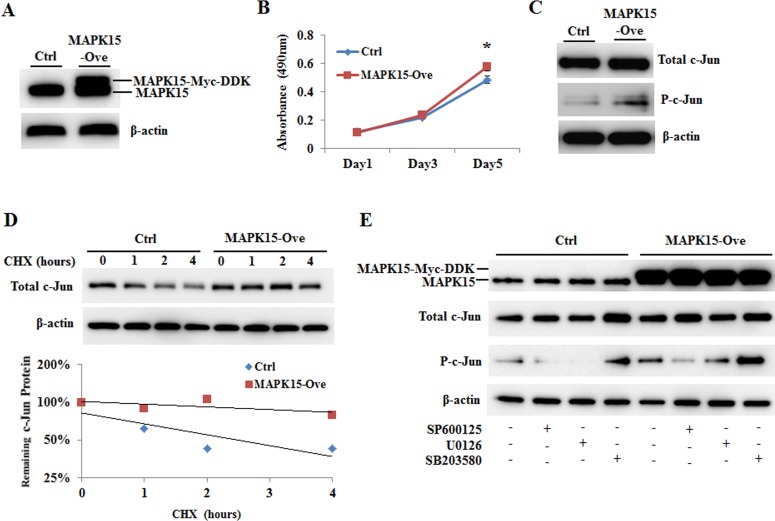
Effect of MAPK15 overexpression on c-Jun phosphorylation AGS cells were transfected with the pCMV6-Entry plasmid that express Myc-DDK tagged MAPK15 protein (MAPK15-Ove) or the Mock plasmid (Ctrl). **A.** MAPK15 protein levels were measured by immunoblot analysis on the 3^rd^ day post-transfection. **B.** Cell proliferation was detected by MTS assay. Absorbance at 490 nm was measured on the 1^st^, 3^rd^, and 5^th^ day post-transfection. Error bars indicate standard deviation (*n* = 4, *P < 0.05). **C.** The protein levels of c-Jun and P-c-Jun were detected by immunoblot analysis on the 3^rd^ day post-transfection. **D.** AGS cells were treated with cycloheximide (80 μg/ml) for 0, 1, 2, or 4 hours, and the c-Jun protein level was measured by immunoblot analysis. The experiment was performed twice, and a similar result was obtained. **E.** c-Jun and phosphorylated-c-Jun levels were analyzed by western blotting after treating AGS cells with SP600125, U0126, or SB203580 as described in the Materials and Methods.

To confirm the data of *MAPK15* knockdown, AGS cells with low copy number were transfected by MAPK15-pCMV6-Myc-DDK. Overexpression of transfected MAPK15-pCMV6-Myc-DDK was monitored by western blotting (Figure 5A). Overexpression of MAPK15 increased cell proliferation (Figure 5B), phosphorylation of c-Jun (Figure 5C) and stability (Figure 5D). Based on these observations, it is likely that MAPK15 regulates c-Jun levels by increasing c-Jun stability through c-Jun phosphorylation.

### MAPK15-dependent phosphorylation of c-Jun

c-Jun N-terminal phosphorylation at Ser63 and Ser73 is required for oncogenic transformation by ras and fos. The phosphorylation can be mediated by JNK, p38, or ERK pathways. To exclude if c-Jun phosphorylation at Ser73 was mediated by the pathways, we analyzed c-Jun phosphorylation through the use of inhibitors of JNK (SP600125), MEK1/2 (U0126), and for p38 (SB203580). The levels of phosphorylated-c-Jun increased irrespective of the treatment of SP600125 (lane 2 *vs*. lane 6), U0126 (lane 3 *vs*. lane 7), and SB203580 (lane 4 *vs*. lane 8) (Figure 5E), suggesting that c-jun phosphorylation by MAPK15 may occur independent of JNK, MEK1/2, and p38.

### Clinicopathological characteristics of MAPK15 CNAs

CNAs data were available in 88 tissues that were used for aCGH and its validation. The association amongst *MAPK* copy number gains in the 88 gastric cancer patients and the clinicopathological characteristics of these patients are shown in Table 1. Copy number gains were found in 15 (17%) of 88 gastric cancers. No significant association between *MAPK15* copy number gains and patient age, sex, tumor location, differentiation, pathologic stage, and family history were observed. Copy number gains were found at a higher prevalence in intestinal type than in diffuse type (27% *vs*. 9%), but the difference was not statistically significant (*P* = 0.07, Fisher's exact test).

**Table 1:** Clinicopathologic characteristics (*N* = 88)

Variables	MAPK15 CNAs		*P* -value
	No (N = 73)	Yes (N = 15)	
Age^a^	58 ± 10	59 ± 13	0.85
Sex			
Men	44	11	
Women	29	4	0.40^c^
Location			
Lower	41	8	
Middle	23	5	
Upper	8	2	
Whole	1	0	0.94
Differentiation^b^			
Differentiated	21	3	
Undifferentiated	31	9	0.51^c^
Lauren classification			
Intestinal	27	10	
Diffuse	42	4	
Mixed	4	1	0.07^c^
T stage			
T1	41	5	
T2	22	7	
T3	9	2	
T4	1	1	0.18^c^
N stage			
N0	44	10	
N1	21	3	
N2	5	1	
N3	3	1	0.87^c^
AJCC stage			
IA	36	6	
1B	13	3	
II	11	2	
IIIA	6	2	
IIIB	3	0	
IV	4	2	0.77^c^
Family history			
No	17	2	
Yes	56	13	0.51^c^

a Mean ± standard deviation

b Differentiation data are missing for 24 patients

c Based on Fisher's exact test

### MAPK15 overexpression occurred at a premalignant stage of gastric mucosa

**Figure 6:**
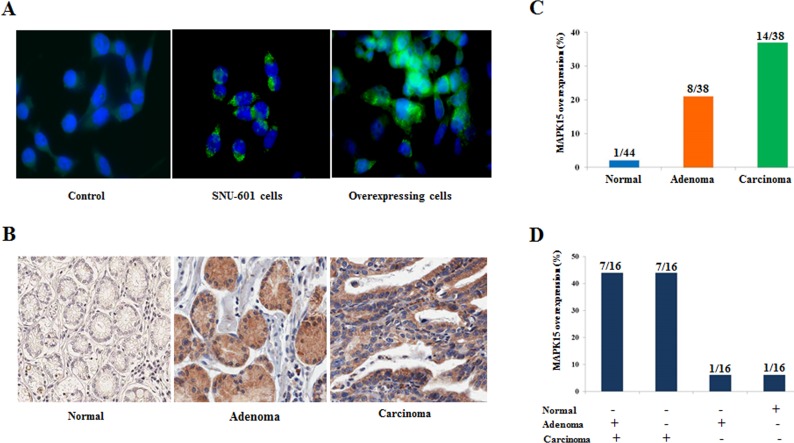
Immunohistochemical staining of MAPK15 **A.** Anti-MAPK15 antibody was tested using immunofluorescence on control, SNU-601 cells, and on AGS cells transfected with MAPK15-pCMV6-Myc-DDK. Cultured cells were stained with anti-MAPK15 antibody. Alexa Fluor-488 goat anti-rabbit secondary antibody was used for fluorescence labeling of MAPK15. DNA was stained with DAPI. **B.** Protein levels of MAPK15 in concurrent legions (normal, adenoma, and carcinoma) from 45 gastric cancer patients were analyzed by immunohistochemistry. The MAPK15 is weakly stained in the cytoplasm. (X200). **C.** The prevalence of MAPK15 overexpression was calculated in normal, adenoma, and carcinoma legions from 45 patients. One patient did not have normal lesion, and some of patients did not have adenoma or carcinoma lesions, and therefore the total number of adenoma and carcinoma lesions was not 45. **D.** The expression status of MAPK15 was compared in concurrent legions individually, to assess the effect of *MAPK15* overexpression on malignant transformation of the stomach. The overexpression of MAPK15 was found in 16 patients, and seven (44%) of the patients have MAPK15 overexpression in concurrent adenoma and carcinoma lesions, and 7 (44%) have MAPK15 overexpression only in carcinoma lesions.

We analyzed the expression of MAPK15 using immunohistochemistry in concurrent lesions of normal, adenoma, and carcinoma derived from 45 gastric cancer patients, to understand the role of MAPK15 in the development of gastric cancer. MAPK15 antibody used was validated by testing it on control, SNU-601, and on AGS cells transfected with MAPK15-pCMV6-Myc-DDK (Figure 6A). MAPK15 overexpression was found in 1 (2%) of 44 normal regions, 8 (21%) of 38 adenomas and in 14 (37%) of 38 carcinoma regions (Figure 6B-6D). A total of 16 patients were found to have MAPK15 overexpression. Seven (44%) of the 16 patients have MAPK15 overexpression in concurrent adenoma and carcinoma lesions, and 7 (44%) have MAPK15 overexpression only in carcinoma lesions (Figure 6D). These results suggest that overexpression of MAPK15 may contribute to the malignant transformation of gastric mucosa. The relationship between MAPK15 overexpression in 38 carcinoma lesions with clinicopathological characteristics was analyzed. However, no association was found between MAPK15 overexpression and patient's age, sex, tumor location, differentiation, and family history (Supplementary Table 2). MAPK15 overexpression tended to occur more frequently in intestinal type than in diffuse type (47% vs. 25%, *P* = 0.13, Fisher's exact test).

## DISCUSSION

Chromosome 8q24 is the commonly amplified region in human cancer, including prostate, breast, colon, and stomach cancer [6, 26, 27]. We also detected frequent DNA copy number gains at 8q24 in gastric cancer. Especially 8q24.21 and 8q24.3 were more frequently amplified than other regions, implying that DNA copy number gains at 8q24.21 or 8q24.3 may play an important role in the development of gastric cancer. Amplification at 8q24.21 is significant because the oncogene *MYC* harbored in this region is known to be associated with many cancers, including gastric cancer [28]. Gains at 8q24.3 are large, spanning genes up to several multiples of ten, many of which are unlikely to be involved in oncogenesis. Copy number gains of *MAPK15* were found in 15 (17%) of 88 gastric cancer tissues and in 7 (44%) of 16 gastric cancer cell lines. There was a significant correlation between the DNA copy number and the mRNA levels.

Knockdown of *MAPK15* by siMAPK15 in this study resulted in inhibition of cell proliferation. However, less is known about the exact molecular mechanisms involved in MAPK15-mediated cell proliferation, as it does not share many characteristics of conventional MAPKs such as the extracellular signal-regulated kinases 1/2 (ERK1/2), c-Jun amino (N)-terminal kinases 1/2/3 (JNK1/2/3), p38 isoforms, and ERK5. While no *in vivo* MAPK15 substrates have been identified thus far, overexpression of MAPK15 is known to increase the transactivation of activator-proteins-1 by phosphorylating c-Jun at Ser63 and Ser73 in colorectal cancer cells [24]. The residues are known to be phosphorylated by JNK/SAPKs in response to UV irradiation and other stress stimuli. In this study, we found that MAPK15 promoted c-Jun phosphorylation independent of JNK, MEK1/2, and p38 in gastric cancer cells (Figure 5E).

c-Jun is the instrumental transcriptional factor in cell growth and cell transformation [29]. Fibroblasts derived from *c-Jun*^−/−^ mouse fetuses exhibit a severe defect in proliferation and result in inefficient G_1_-to-S phase progression [30]. Cancer cells appear to have several ways to maintain a high level of c-Jun. Constitutively active MAP kinase signaling in cancer cells can result in a hyper-phosphorylation of c-Jun at its N-terminus to protect c-Jun from degradation [31, 32]. Knockdown of *MAPK15* by siMAPK15 in this study resulted in cell cycle arrest at G_1_/S phase and in the reduction of c-Jun phosphorylation. Additionally, treatment of SNU601 cells with 10 μM cyclohexamide, an inhibitor of protein biosynthesis in eukaryotic organisms, substantially decreased total c-Jun amounts in a time-dependent manner in cells transfected with siMAPK15 rather than in those with transfected with siCtrl. The effects of siMAPK15 on cell proliferation, c-Jun phosphorylation, stability (Supplementary Figure 5), and cell cycle (Supplementary Figure 6) were also found in different cell lines: SNU-216 with copy number gains and SNU-638 and MKN-45 without copy number gains. Based on these observations, MAPK15 may regulate c-Jun levels by increasing the phosphorylation and stability of c-Jun in gastric cancer cells.

We analyzed the expression of MAPK15 in normal, adenoma, and carcinoma tissues derived from the same gastric cancer patients in order to determine the time point of MAPK15 expression during the process of malignant transformation. MAPK15 overexpression was found in 2% of normal tissues, 21% of adenoma, and 37% of carcinoma tissues. Seven (44%) of the 16 patients with MAPK15 overexpression have the overexpression in concurrent adenoma and carcinoma lesions. This finding suggests that patients with MAPK15 overexpression in benign tumor may be susceptible to the progression of the disease. Copy number gains or overexpression of MAPK15 in premalignant lesion of gastric mucosa can be a risk factor of gastric cancer. Therefore, those patients need careful monitoring for the prevention and early detection of gastric cancer.

*MYC* is a potent oncogene. Amplification of 8q24.21, containing *MYC* locus, shows high frequency in cancer and this locus is close to 8q24.3, which contains the *MAPK15* gene. Therefore, there is a possibility that gene dosage increase of MAPK15 in this study may affected by amplification of *MYC*. To rule out the possibility, we analyzed the prevalence of co-amplification of *MAPK15* and *MYC*. Based on aCGH data, copy number gains of *MYC* (8q24.21) and *MAPK15* (8q24.3) were found in 12 and 8 of 40 gastric cancers, respectively. In addition, seven (88%) of 8 cancers with copy number gains of *MAPK15* also had copy number gains of *MYC*. Therefore, it is possible that gene dosage increase of *MAPK15* may be partly influenced by *MYC* amplification. However, further study is required to clearly understand the effect of *MYC* amplification on gene dosage of *MAPK15* because *MAPK15* is located to a region 1.6 Mb distal to the *MYC* locus.

In spite of frequent overexpression of MAPK15 in human cancers, mechanisms underlying the overexpression are not clear in gastric cancer. The overexpression and CNAs of *MAPK15* was found in 14 (37%) of 38 formalin-fixed paraffin-embedded tissues and in 15 (17%) of 88 fresh-frozen gastric cancer tissues, respectively. Although there is no report on the mutation of *MAPK15* in gastric cancer to date, our data suggest that MAPK15 overexpression may result from copy number gains as well as other molecular alterations in gastric cancer. In this study, three out of seven cell lines with copy number gain did not exhibit increased *MAPK15* expression at mRNA level (Figure 3D). In addition, no significant correlation between *MAPK15* mRNA and protein levels in 16 gastric cancer cell lines was observed (Supplementary Figure 3). Based on these observations, MAPK1*5* expression in vitro may be regulated by post-transcriptional and/or post-translationtional mechanisms because copy number gain of *MAPK15* was correlated with an increase of MAPK15 expression in vivo (Supplementary Figures 1 and 2).

In conclusion, the findings from the present study suggested that *MAPK15* copy number gains may occur at a premalignant stage of gastric cancer and contribute to malignant transformation of gastric mucosa by increasing the phosphorylation and stability of c-Jun. In addition, MAPK15 overexpression in premalignant gastric lesion may be a predictive biomarker for the progression to invasive gastric cancer.

## MATERIALS AND METHODS

### Study population and DNA extraction

A total of 133 patients who underwent curative surgical resection for gastric cancer between February 2003 and November 2012 at the Department of Surgery in the Samsung Medical Center, Seoul, Korea, participated in this study. The patients consisted of 86 men and 47 women, and the mean age of the patients was 59 at diagnosis of the disease. Seventy-one patients were classified as diffuse type, 55 as intestinal type, and 7 as a mixed type according to the Lauren classification. The surgically removed tumor tissues were collected after obtaining permission from the appropriate Institutional Review Board and written informed consent from all the study patients. The tumors were staged using TNM classification established by the American Joint Committee on Cancer (AJCC, 6^th^ edition).

### Multiplex ligation-dependent probe amplification (MLPA) analysis

MLPA analysis was performed using the SALSA MLPA kit P200 (MRC-Holland, Amsterdam, Netherlands) according to the manufacturer's instructions [33]. P200 kit contains 14 internal control probes to assess DNA denaturation and DNA quantity, and also for the X and Y chromosome. The MLPA primers of *MAPK15* were 5′-GGG TTC CCT AAG GGT TGG ATC CAA TGT GCT CCT GGA TGC CAA CTG-3′ (forward) and 5′-CAC AGT GAA GCT GTG TGA CTT TGG CCT GGT TCT CTA GAT TGG ATG TTG CTG GCA C-3′ (reverse). DNA samples were diluted with TE to 5 μl and were heated at 98°C for 5 min in PCR tubes, in a thermocycler with a heated lid. After addition of 1.5 μl MLPA buffer and 1.5 μl probe mix, samples were further heated for 1 min at 95°C and then incubated for 16 h at 60°C. Ligation of annealed oligonucleotides was performed by diluting the samples to 40 μl with dilution buffer containing 1 U Ligase-65 enzyme, and incubated for 15 min at 54°C. The ligase enzyme was inactivated by heating at 98°C for 5 min and ligation products were amplified by PCR. Ten μl of a buffered solution containing the PCR primers, dNTPs and SALSA polymerase (MRC-Holland, Amsterdam, Netherlands) were added at 60°C. PCR was carried for 35 cycles (30 s at 95°C, 30 s at 60°C and 1 min at 72°C). The MLPA PCR reactions were separated on a capillary electrophoresis system ABI-Prism 3130 (Applied Biosystems, Foster City, CA), and the data was analyzed using the GeneMaker 2.0.0 (SoftGenetics, State College, PA). Probe ratios of above 1.3 or below 0.7 were regarded as indicative of a gain or loss, respectively.

### DNA copy number analysis by quantitative real-time PCR (qPCR)

DNA copy number of *MAPK15* in 16 gastric cancer cell lines and 48 gastric cancers were detected using commercially available, predesigned TaqMan Copy Number Assay (Assay ID: Hs00233516_cn, consisting of a pair of unlabeled primers and a FAM labeled MGB probe) and *RNase P* Copy Number Reference Assay (consisting of a pair of unlabeled primers and a VIC-labeled TAMRA probe) (Applied Biosystems) as previously described [34, 35]. Genomic DNA was isolated using a DNA Mini Kit (Qiagen, Valencia, CA) for cell lines. The tissue sections were placed on slides and stained with H&E to evaluate the admixture of tumorous and non-tumorous tissues, before DNA was extracted from the fresh-frozen tissues. Tumor and non-tumorous areas were carefully microdissected under a microscope. The microdissected tissues were digested with proteinase K, and genomic DNA was isolated using DNeasy tissue kit (Qiagen, Valencia, CA) according to the manufacturer's instructions. Quantitative real-time polymerase chain reaction (qPCR) was carried out in triplicate on an ABI 7900HT instrument (Applied Biosystems). The thermal cycling conditions were 95°C for 10 min followed by 40 cycles of 95°C for 15 s and 60°C for 1 min. Analyses of the qPCR data were performed using the Copy Caller v2.0 software (Applied Biosystems).

### Quantitative reverse transcription PCR (qRT-PCR)

Total RNA was isolated using PureLink RNA Mini Kit (Invitrogen, Carlsbad, CA), and RT-PCR was carried out using the SuperScript VILO cDNA Synthesis Kit (Invitrogen, Carlsbad, CA) according to the manufacturer's protocol. Real-time PCR was performed with SYBR green dye (Qiagen, Valencia, CA) using the ABI PRISM 7900HT Sequence Detection System (Applied Biosystems, Foster City, CA) at the following conditions: an initial denaturation step at 95 °C for 10 min, followed by 40 cycles at 95 °C for 15 s and 60 °C for 30 s. The PCR primer sequences used for qRT-PCR are shown in Supplementary Table 1. GAPDH or RPLP0 was used to normalize the target mRNA level in each sample.

### Cell culture and transient transfection assay

Human gastric cancer cell lines were purchased from the Korean Cell Line Bank (Seoul National University College of Medicine, Seoul, Korea) in September 2013 and authenticated using real-time PCR in October 2013. The cells were grown in Roswell Park Memorial Institute Medium 1640 (RPMI1640) (Lonza, Allendale, NJ), supplemented with 10% fetal bovine serum (FBS), 100 U of penicillin/mL, and 100 μg of streptomycin/mL at 37°C in a 5% CO_2_ atmosphere. Three kinds of siRNA sequences were tested for *MAPK15* knockdown: CUU UUA GGG AUA AGA CAG A (siMAPK15#1), CGG AGG AUG UUC AGC ACC U (siMAPK15#2), and AGC CGU CCA AUG UGC UCC U (siMAPK15#3). Transfection of MAPK15-specific siRNA (siMAPK15, BioNeer, DaeJeon, Korea) or nonspecific siRNA (siCtrl, BioNeer) was carried out using Lipofectamine RNAiMAX (Invitrogen, Carlsbad, CA) according to the manufacturer's protocol. Total RNA or protein was harvested at the indicated times after siRNA transfection. Lipofectamine 2000 (Invitrogen, Carlsbad, CA) was used for MAPK15 overexpression. Transfection of pCMV6-Entry vector (OriGene, Rockville, MD) that expresses Myc-DDK tagged MAPK15 was carried out when cells reached 80∼90% confluence. Total protein was harvested, and the tagged MAPK15 protein was detected by immunoblot analysis.

### Inhibition of MAPK15 ser73 phosphorylation

AGS cells with low copy number were transfected with the pCMV6-Entry vector (0.8 μg/ml) that expresses Myc-DDK tagged MAPK15 protein or mock. On the 3^rd^ day post-transfection, the cells were treated with 40μM JNK inhibitor SP600125 (Sigma) for 1 hour, 20μM MEK1/2 inhibitor U0126 (Sigma) for 2 hours, or 20μM p38 inhibitor SB203580(sigma) for 2 hours. After stimulation with culture medium including 20% FBS for 30 minutes, total protein was harvested and c-Jun and phosphorylated-c-Jun levels were detected by western blot analysis.

### Immunoblot analysis

Total proteins were extracted from the cultured cells using a lysis buffer containing Protease Inhibitor Cocktail (Roche Applied Science, Indianapolis, IN). The lysates were heated to 95°C for 5 min, loaded on 10% sodium dodecyl sulfate-polyacrylamide gels and transferred to membranes (Millipore, MA). After blocking with a 3% solution of fetal bovine serum, the membranes were probed with antibodies directed against MAPK15 (ab128007, Abcam, Cambridge, UK or H-22, sc-130814, Santa Cruz Biotechnology, Santa Cruz, CA) or phosphorylated-MAPK15 (T175/Y177) (ab73209, Abcam, Cambridge, UK) or c-Jun (60A8, #9165, Cell Signaling, Danvers, MA) or phosphorylated-c-Jun (#9164, Cell Signaling) or β-actin (13E5, #4970, Cell Signaling). The membranes were then incubated with horseradish peroxidase-conjugated secondary antibodies (Cell Signaling) and visualized with the Immun-Star Western Kit (Bio-Rad, Hercules, CA) according to the manufacturer's instructions.

### Cell proliferation assay

Gastric cancer cell lines were cultured in 24-well plates (5 × 10^4^ cells/well) and transfected with siRNA (40nM) or pCMV6-Entry vector (0.8 μg/ml). Cell proliferation was measured on the 1^st^, 3^rd^ and 5^th^ day after siRNA transfection by MTS [3-(4,5-dimethylthiazol-2yl)-5-(3-carboxymethoxyphenyl)-2-(4-sulphophenyl)-2H-tetrazolium] assay as decribed previously [36]. One hundred-microliters of CellTiter 96^®^ AQueous One Solution (Promega, Madison, WI) were added to each well and cells were incubated for 1 hour in a 37°C, 5% CO_2_ incubator. Absorbance at 490 nm was measured in a microplate reader (Bio-Rad, Hercules, CA).

### Cell cycle analysis

Gastric cancer cell lines were cultured in 6-well plates and transfected with siMAPK15, siCtrl, or pCMV6-Entry vector. Cells were collected on the 3^rd^ day after siRNA transfection and centrifuged at 1200 revolutions per minute for 5 minutes. The cells were resuspended, washed twice in phosphate-buffered saline (PBS), and fixed in 70% ice-cold (−20°C) ethanol. The cells were then spun down and washed in PBS that contained 0.5% bovine serum albumin. Cell cycle was analyzed using the FITC BrdU Flow Kit (#559619, BD, Franklin Lakes, NJ) or propidium iodide (PI) solution containing 50μg/mL PI according to the manufacturer's protocol. Briefly, on the 3^rd^ day post-siRNA transfection, SNU-601 cells were treated with 10 μM BrdU for 2 hours, and collected. The cells were incubated with a FITC-conjugated anti-BrdU antibody. Total DNA was stained with 7-AAD. SNU-638 and MKN-45 cells were stained using PI solution. Stained cells were analyzed using a FACS Calibur system (BD, Franklin Lakes, NJ) and CELLQuest software (version 3.3; Becton Dickinson).

### Immunohistochemistry

To test anti-MAPK15 antibody, SNU-601 cells and AGS cells transfected with MAPK15-pCMV6-Myc-DDK plasmid were fixed with 4% PFA, and the antigen was retrieved by heating at 100°C for 10 minutes in sodium citrate buffer (10mM Sodium Citrate, 0.05% Tween 20, pH 6.0). The cells were stained with anti-MAPK15 antibody (ab128007, Abcam, Cambridge, UK) and Alexa Fluor-488 goat anti-rabbit secondary antibody. The DNA was stained with DAPI. Images were obtained by immunofluorescence microscopy Zeiss AX10 (Zeiss, Gottingen, Germany). Tissue microarrays (TMAs) of concurrent 3 lesions (normal, adenoma, and carcinoma) from 45 gastric cancer patients and immunohistochemical staining of MAPK15 were conducted using conventional techniques. Briefly, representative areas were carefully selected from a donor paraffin-embedded tissue block using H&E staining, and 3 tissue cores (1.5 mm in diameter) from each region were then transferred to a recipient paraffin wax block using a Tissue Microarrayer (Beecher Instruments, Silver Spring, MD). Three-micrometer-thick sections from paraffin-embedded gastric cancer tissue blocks were deparaffinized in xylene and rehydrated through a series of alcohols, and washed in phosphate-buffered saline. The sections were subjected to antigen-retrieval by autoclaving in Trilogy antigen retrieval solution (Cell Marque, Austin, TX) in a microwave oven for 20 min. After cooling to room temperature, the tissue sections were treated with 5% H_2_O_2_ in methanol for 10 min to block endogenous peroxidase activity. Nonspecific immunostaining was suppressed with 2% low-fat milk in PBS for 30 min. The sections were then incubated overnight at 4 °C with polyclonal anti-MAPK15 antibody (ab128007, Abcam, Cambridge, UK). A negative control for each tissue section was made by omitting the primary antibody. Detection of immunoreactivity was done using Vectastain Elite ABC reagent (Vector Laboratories); 3.3′-diaminobenzidine tetrahydrochloride was used as a chromogen and hematoxylin was used as the nuclear counter stain.

### Interpretation of MAPK15 expression

All available slides were reviewed by 2 authors (K-M.K and D-H.K), who were blinded to all clinicopathological variables, to reduce inter-observer variability. Semi-quantitative assessment of immunoreactivities for MAPK1*5* was performed by evaluation of the percentage of positively stained cells and staining intensity in the cytoplasm alone. The overall index score for each sample was determined by the addition of the intensity score (0, none; 1, weak; 2, moderate; 3, strong) and a proportion score of positively stained tumor cells (0, absent; 1, 0-10%; 2, 10-50%; 3, 50-80%; 4, >80%). MAPK15 was considered to be overexpressed if the mean index scores of MAPK15 expression in cytoplasm for each region were ≥ 2.

### Statistical analysis

The t-test (or Wilcoxon rank-sum test) and Pearson's chi-square test (or Fisher's exact test) were used to analyze continuous and categorical variables between two groups by univariate analysis, respectively. Correlation between DNA copy number and mRNA levels was analyzed by Pearson's (or Spearman's) correlation coefficients. All statistical analyses were 2-sided, with a 5% type I error rate.

## SUPPLEMENTARY FIGURES AND TABLES



## References

[1] Brenner H, Rothenbacher D, Arndt V (2009). Epidemiology of stomach cancer. Methods Mol Biol.

[2] Shlien A, Malkin D (2010). Copy number variations and cancer susceptibility. Curr Opin Oncol.

[3] Park CH, Rha SY, Jeung HC, Kang SH, Ki DH, Lee WS, Noh SH, Chung HC (2010). Identification of novel gastric cancer-associated CNVs by integrated analysis of microarray. J Surg Oncol.

[4] El-Rifai W, Sarlomo-Rikala M, Andersson LC, Knuutila S, Miettinen M (2000). DNA sequence copy number changes in gastrointestinal stromal tumors: tumor progression and prognostic significance. Cancer Res.

[5] Kokkola A, Monni O, Puolakkainen P, Nordling S, Haapiainen R, Kivilaakso E, Knuutila S (1998). Presence of high-level DNA copy number gains in gastric carcinoma and severely dysplastic adenomas but not in moderately dysplastic adenomas. Cancer Genet Cytogenet.

[6] Tsukamoto Y, Uchida T, Karnan S, Noguchi T, Nguyen LT, Tanigawa M, Takeuchi I, Matsuura K, Hijiya N, Nakada C, Kishida T, Kawahara K (2008). Genome-wide analysis of DNA copy number alterations and gene expression in gastric cancer. J Pathol.

[7] Cheng L, Wang P, Yang S, Yang Y, Zhang Q, Zhang W, Xiao H, Gao H, Zhang Q (2012). Identification of genes with a correlation between copy number and expression in gastric cancer. BMC Med Genomics.

[8] Lee KY, Liu YH, Ho CC, Pei RJ, Yeh KT, Cheng CC, Lai YS (2004). An early evaluation of malignant tendency with plectin expression in human colorectal adenoma and adenocarcinoma. J Med.

[9] Ho JC, Cheung ST, Patil M, Chen X, Fan ST (2006). Increased expression of glycosyl-phosphatidylinositol anchor attachment protein 1 (GPAA1) is associated with gene amplification in hepatocellular carcinoma. Int J Cancer.

[10] Jung J, Kim JM, Park B, Cheon Y, Lee B, Choo SH, Koh SS, Lee S (2010). Newly identified tumor-associated role of human Sharpin. Mol Cell Biochem.

[11] Killian A, Sarafan-Vasseur N, Sesboue R, Le Pessot F, Blanchard F, Lamy A, Laurent M, Flaman JM, Frébourg T (2006). Contribution of the BOP1 gene, located on 8q24, to colorectal tumorigenesis. Genes Chromosomes Cancer.

[12] Jin X, Moskophidis D, Mivechi NF (2011). Heat shock transcription factor 1 is a key determinant of HCC development by regulating hepatic steatosis and metabolic syndrome. Cell Metab.

[13] Zhang Y, Bharadwaj U, Logsdon CD, Chen C, Yao Q, Li M (2010). ZIP4 regulates pancreatic cancer cell growth by activating IL-6/STAT3 pathway through zinc finger transcription factor CREB. Clin Cancer Res.

[14] Maire G, Yoshimoto M, Chilton-MacNeill S, Thorner PS, Zielenska M, Squire JA (2009). Recurrent RECQL4 imbalance and increased gene expression levels are associated with structural chromosomal instability in sporadic osteosarcoma. Neoplasia.

[15] Nagasaka K, Pim D, Massimi P, Thomas M, Tomaic V, Subbaiah VK, Kranjec C, Nakagawa S, Yano T, Taketani Y, Myers M, Banks L (2010). The cell polarity regulator hScrib controls ERK activation through a KIM site-dependent interaction. Oncogene.

[16] Matsushita K, Tomonaga T, Kajiwara T, Shimada H, Itoga S, Hiwasa T, Kubo S, Ochiai T, Matsubara H, Nomura F (2009). c-myc suppressor FBP-interacting repressor for cancer diagnosis and therapy. Front Biosci.

[17] Yu M, Schreek S, Cerni C, Schamberger C, Lesniewicz K, Poreba E, Vervoorts J, Walsemann G, Grötzinger J, Kremmer E, Mehraein Y, Mertsching J (2005). PARP-10, a novel Myc-interacting protein with poly(ADP-ribose) polymerase activity, inhibits transformation. Oncogene.

[18] Abe MK, Saelzler MP, Espinosa R, Kahle KT, Hershenson MB, Le Beau MM, Rosner MR (2002). ERK8, a new member of the mitogen-activated protein kinase family. J Biol Chem.

[19] Bogoyevitch MA, Court NW (2004). Counting on mitogen-activated protein kinases—ERKs 3, 4, 5, 6, 7 and 8. Cell Signal.

[20] Coulombe P, Meloche S (2007). Atypical mitogen-activated protein kinases: structure, regulation and functions. Biochim Biophys Acta.

[21] Iavarone C, Acunzo M, Carlomagno F, Catania A, Melillo RM, Carlomagno SM, Santoro M, Chiariello M (2006). Activation of the Erk8 mitogen-activated protein (MAP) kinase by RET/PTC3, a constitutively active form of the RET proto-oncogene. J Biol Chem.

[22] Santoro M, Melillo RM, Carlomagno F, Vecchio G, Fusco A (2004). Minireview: RET: normal and abnormal functions. Endocrinology.

[23] Groehler AL, Lannigan DA (2010). A chromatin-bound kinase, ERK8, protects genomic integrity by inhibiting HDM2-mediated degradation of the DNA clamp PCNA. J Cell Biol.

[24] Xu YM, Zhu F, Cho YY, Carper A, Peng C, Zheng D, Yao K, Lau AT, Zykova TA, Kim HG, Bode AM, Dong Z (2010). Extracellular signal-regulated kinase 8-mediated c-Jun phosphorylation increases tumorigenesis of human colon cancer. Cancer Res.

[25] Cerone MA, Burgess DJ, Naceur-Lombardelli C, Lord CJ, Ashworth A (2011). High-throughput RNAi screening reveals novel regulators of telomerase. Cancer Res.

[26] Beroukhim R, Mermel CH, Porter D, Wei G, Raychaudhuri S, Donovan J, Barretina J, Boehm JS, Dobson J, Urashima M, Mc Henry KT, Pinchback RM (2010). The landscape of somatic copy-number alteration across human cancers. Nature.

[27] Ahmadiyeh N, Pomerantz MM, Grisanzio C, Herman P, Jia L, Almendro V, He HH, Brown M, Liu XS, Davis M, Caswell JL, Beckwith CA, Hills A, Macconaill L, Coetzee GA, Regan MM, Freedman ML (2010). 8q24 prostate, breast, and colon cancer risk loci show tissue-specific long-range interaction with MYC. Proc Natl Acad Sci U S A.

[28] Kim YJ, Ghu HD, Kim DY, Kim HJ, Kim SK, Park CS (1993). Expression of cellular oncogenes in human gastric carcinoma: c-myc, c-erb B2, and c-Ha-ras. J Surg Oncol.

[29] Ip YT, Davis RJ (1998). Signal transduction by the c-Jun N-terminal kinase (JNK)—from inflammation to development. Curr Opin Cell Biol.

[30] Schreiber M, Kolbus A, Piu F, Szabowski A, Möhle-Steinlein U, Tian J, Karin M, Angel P, Wagner EF (1999). Control of cell cycle progression by c-Jun is p53 dependent. Genes Dev.

[31] Musti AM, Treier M, Bohmann D (1997). Reduced ubiquitin-dependent degradation of c-Jun after phosphorylation by MAP kinases. Science.

[32] Zhang J, Zhu F, Li X, Dong Z, Xu Y, Peng C, Li S, Cho YY, Yao K, Zykova TA, Bode AM, Dong Z (2012). Rack1 protects N-terminal phosphorylated c-Jun from Fbw7-mediated degradation. Oncogene.

[33] Schouten JP, McElgunn CJ, Waaijer R, Zwijnenburg D, Diepvens F, Pals G (2002). Relative quantification of 40 nucleic acid sequences by multiplex ligation-dependent probe amplification. Nucleic Acids Res.

[34] Togashi Y, Arao T, Kato H, Matsumoto K, Terashima M, Hayashi H, de Velasco MA, Fujita Y, Kimura H, Yasuda T, Shiozaki H, Nishio K (2014). Frequent amplification of ORAOV1 gene in esophageal squamous cell cancer promotes an aggressive phenotype via proline metabolism and ROS production. Oncotarget.

[35] Lorenzetto E, Brenca M, Boeri M, Verri C, Piccinin E, Gasparini P, Facchinetti F, Rossi S, Salvatore G, Massimino M, Sozzi G, Maestro R (2014). YAP1 acts as oncogenic target of 11q22 amplification in multiple cancer subtypes. Oncotarget.

[36] Shen H, Morrison CD, Zhang J, Underwood W, Yang N, Frangou C, Eng K, Head K, Bollag RJ, Kavuri SK, Rojiani AM, Li Y (2013). 6p22. 3 amplification as a biomarker and potential therapeutic target of advanced stage bladder cancer. Oncotarget.

