# Supplementary material for: Overexpression of MAPK15 in gastric cancer is associated with copy number gain and contributes to the stability of c-Jun
Source: Oncotarget. 2015 May 19;6(24):20190–203. doi: 10.18632/oncotarget.4171 (PMC4652997; doi:10.18632/oncotarget.4171)
Supplement: Supplementary file 1 [file oncotarget-06-20190-s001.pdf]

Overexpression of MAPK15 in gastric cancer is associated with copy number gain and contributes to the stability of c-Jun

Supplementary Material

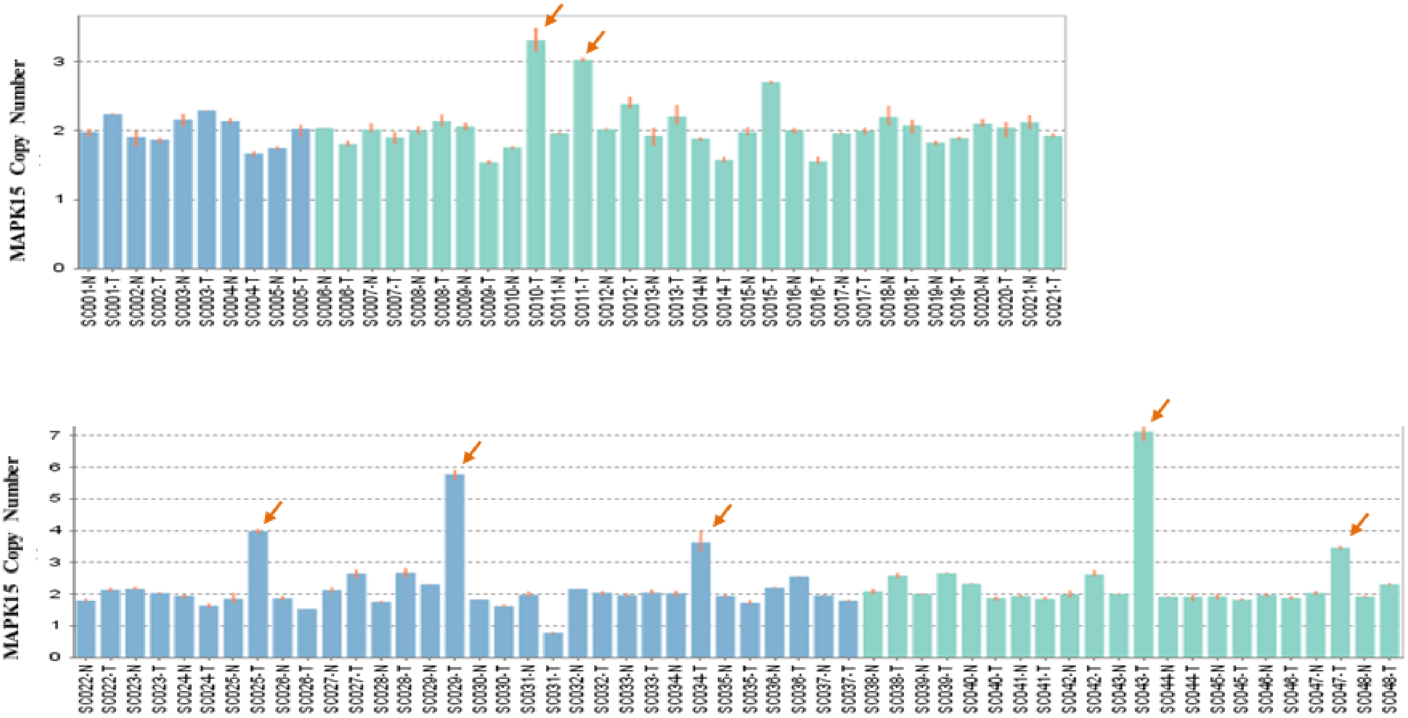

Supplementary Fig. 1. Copy number alterations of *MAPK15*. Copy numbers of *MAPK15* were analyzed using qPCR in tumor and matched normal tissues from 48 gastric cancer patients. The “N” and “T” represent normal and tumor tissues, respectively. Arrows indicate samples with copy number gains of *MAPK15*.

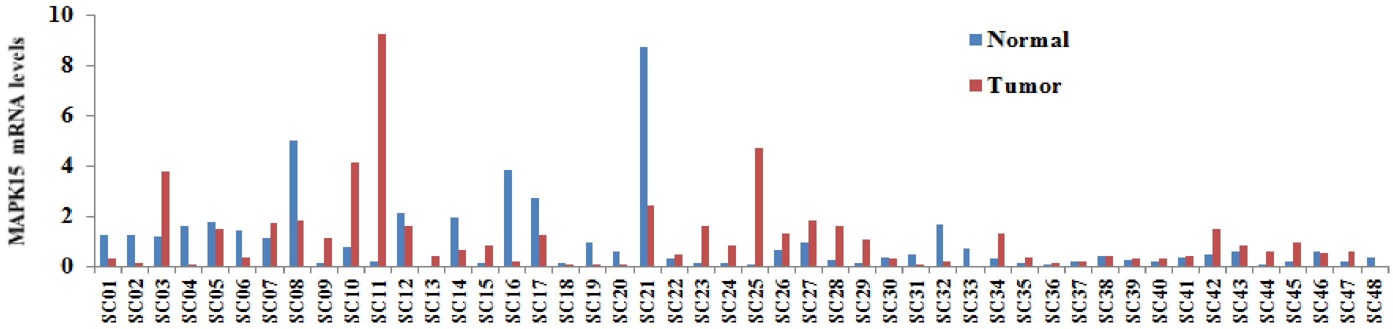

Supplementary Fig. 2. mRNA levels of *MAPK15*. The mRNA levels of *MAPK15* were analyzed using qRT-PCR in tumor and matched normal tissues from 48 gastric cancer patients. The mRNA levels in each sample were normalized to the internal control of RPLP0.

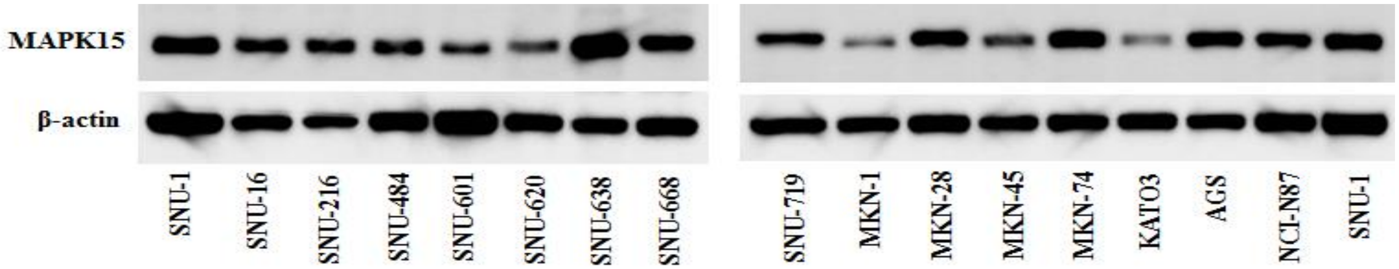

Supplementary Fig. 3. Protein levels of MAPK15 in 16 gastric cancer cell lines

Protein levels of MAPK15 were measured using western blotting in the 16 gastric cancer cell lines as described in the Materials and Methods.

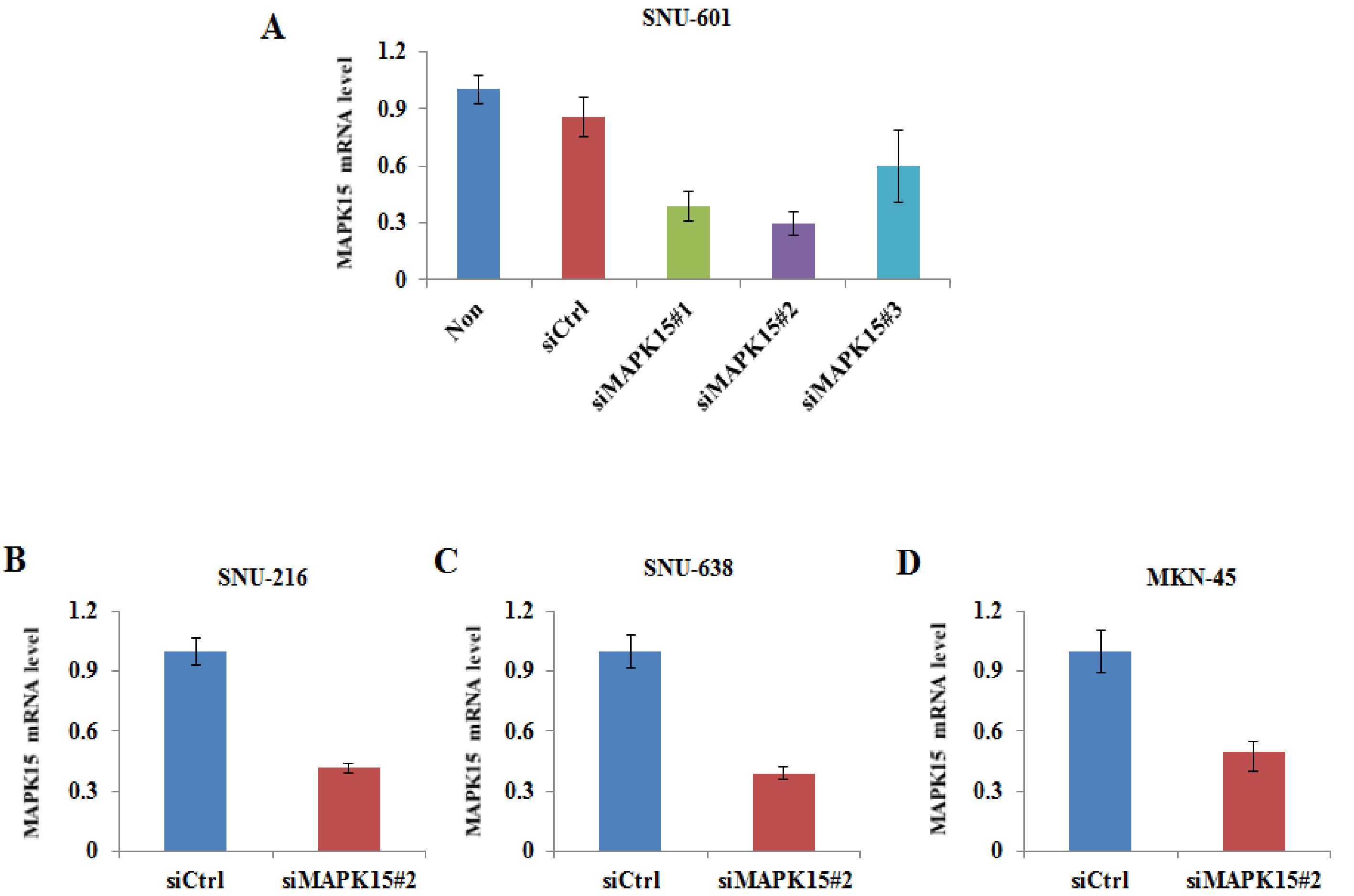

Supplementary Fig. 4. MAPK15 siRNA test

(A) SNU-601 cells were transfected with three MAPK15 siRNAs (siMAPK15#1, #2, #3), nonspecific siRNA (siCtrl), or without (Non). *MAPK15* mRNA levels were measured by qRT-PCR on the 3rd day post-transfection. Error bars indicate standard deviation (n = 3, *P < 0.05). (B) SNU-216, SNU-638, and MKN-45 cells were transfected with the MAPK15 siRNA (siMAPK15#2) or nonspecific siRNA (siCtrl). *MAPK15* mRNA levels were measured by qRT-PCR on the 3rd day post-transfection. Error bars indicate standard deviation (n = 3, *P < 0.05).

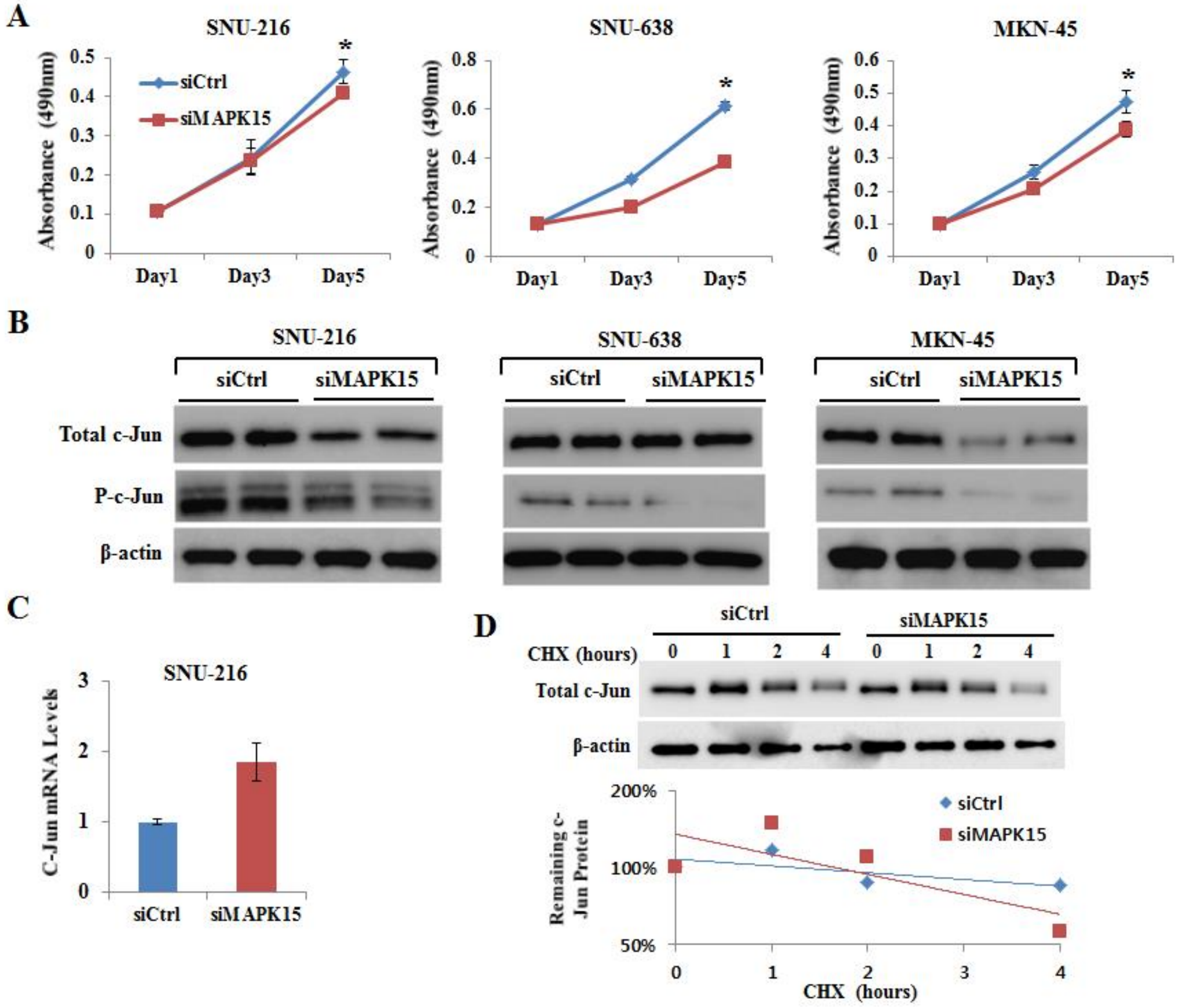

Supplementary Fig. 5. Knockdown of *MAPK15* in other cell lines.

SNU-216, SNU-638 and MKN-45 cells were transfected with MAPK15 siRNA (siMAPK15) or nonspecific siRNA (siCtrl). (A) Cell proliferation was detected by MTS assay. Absorbance at 490 nm was measured on the 1st, 3rd, and 5th day post-transfection. Error bars indicate standard deviation (n = 4, *P < 0.05). (B) c-Jun and P-c-Jun protein levels were detected by immunoblot analysis on the 3rd day post-transfection. The experiment was performed twice and similar results were obtained. (C) *c-Jun* mRNA levels in SNU-216 cells which transfected with siMAPK15 or siCtrl were detected by qRT-PCR. The mRNA level in each sample was normalized to the internal control of GAPDH. Error bars indicate standard deviation (n = 3). (D) On the 3rd day post siRNA transfection, SNU-638 cells were treated with cycloheximide (80μg/ml), a protein synthesis inhibitor, for 0, 1, 2, or 4 hours, and the c-Jun protein level was measured by immunoblot analysis. The experiment was performed twice, and a similar result was obtained.

A

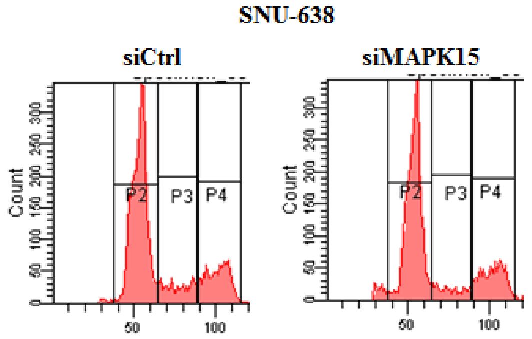

	G0/G1	S	G2/M
siCtrl	66±0.2%	12.1±0.3%	20.2±0.5%
siMAPK15	66.4±0.7%	8.4±0.3%	21.6±0.7%

B

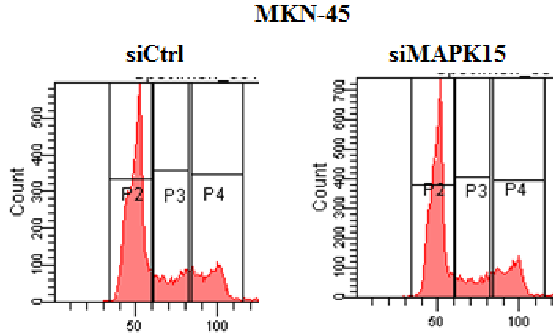

	G0/G1	S	G2/M
siCtrl	62.6±1.6%	15.9±0.4%	19.7±2%
siMAPK15	63.7±1%	14.3±0.9%	20.9±2.4%

Supplementary Fig. 6. Cell cycle analysis. SNU-638 and MKN-45 cells were transfected with MAPK15 siRNA (siMAPK15) or nonspecific siRNA (siCtrl). On the 3rd day post-transfection, DNA profiles of propidium iodide-stained cells were analyzed by FACS Calibur system.

Supplementary Table 1: Primer sequences for quantitative reverse transcription PCR

	Forward	Reverse
MAPK15	5′-ACG TGC GCT CCA TCT TCT AC-3′	5′-ACG TGC GCT CCA TCT TCT AC-3′
c-Jun	5′-CAG GTG GCA CAG CTT AAA CA-3′	5′-GTT TGC AAC TGC TGC GTT AG-3′
GAPDH	5′-ATG TTC GTC ATG GGT GTG AA-3′	5′-TGT GGT CAT GAG TCC TTC CA-3′
RPLP0	5′- CCT TGT CTG TGG AGA CGG ATT AC-3′	5′- GCC AAG AAG GCC TTG ACC TT-3′

Supplementary Table 2: Clinicopathological characteristics (N=38)

Variables	MAPK15 overexpression		P -value
	No (N = 24)	Yes (N = 14)	
Age[a]	59 ± 12	59 ± 11	0.94
Sex			
Men	15	11	
Women	9	3	0.56[c]
Location			
Lower	13	7	
Middle	7	5	
Upper	3	2	
Whole	1	0	0.94[c]
Differentiation[b]			
Differentiated	9	2	
Undifferentiated	12	8	0.13[c]
Lauren classification			
Intestinal	9	8	
Diffuse	14	5	
Mixed	1	1	0.28[c]
Family history			
No	8	3	
Yes	16	11	0.49[c]

[a] Mean ± standard deviation

[b] Differentiation data are missing for 7 patients.

[c] Based on Fisher's exact test
